# Supplementary material for: AnophelesModel: An R package to interface mosquito bionomics, human exposure and intervention effects with models of malaria intervention impact
Source: PLoS Comput Biol. 2024 Sep 13;20(9):e1011609. doi: 10.1371/journal.pcbi.1011609 (PMC11424000; doi:10.1371/journal.pcbi.1011609)
Supplement: S1 Text — (DOCX) [file pcbi.1011609.s001.docx]

AnophelesModel: An R package to interface mosquito bionomics, human exposure and intervention effects with models of malaria intervention impact

Supplementary Text

Monica Golumbeanu, Olivier Briët, Clara Champagne, Jeanne Lemant, Munir Winkel, Barnabas Zogo, Maximilian Gerhards, Marianne Sinka, Nakul Chitnis, Melissa Penny, Emilie Pothin, Tom Smith

Contents

[A. Guidelines for defining input parameters in the package 2](#_Toc175900222)

[A.a Defining the vector bionomics parameters: **def_vector_params()** 2](#_Toc175900223)

[A.b Defining the host-specific parameters: **def_host_params()** 3](#_Toc175900224)

[A.c Defining activity patterns: **def_activity_patterns()** 5](#_Toc175900225)

[A.d Defining intervention effects: **def_intervention_effects()** 5](#_Toc175900226)

[B. Modelling the effects of vector control interventions 6](#_Toc175900227)

[B.a LLIN effects models 8](#_Toc175900228)

[B.b IRS effects models 9](#_Toc175900229)

[B.c Combinations of interventions 11](#_Toc175900230)

[C. Calculating human exposure to mosquitoes 14](#_Toc175900231)

[D. Incorporating parameter uncertainty 16](#_Toc175900232)

[E. Considering multiple mosquito species 17](#_Toc175900233)

[F. Using AnophelesModel to parameterize OpenMalaria 17](#_Toc175900234)

# Guidelines for defining input parameters in the package

To use the package, the user needs to provide information about the mosquito bionomics characteristics, human and mosquito activity patterns, as well as about the effects of vector control interventions. These three layers of data are defined through three function calls described in the following paragraphs.

## A.a Defining the vector bionomics parameters: **def_vector_params()**

Several input, **field measurable bionomics parameters** are used to parameterise the model as described in (1):

- parous rate (M): the proportion of host-seeking mosquitoes that have laid eggs at least once.
- human blood index (Chi): the proportion of blood meals derived from humans by mosquitoes
- sac rate (A0): proportion of mosquitoes who laid eggs the same day
- endophily: proportion of indoor resting mosquitoes
- endophagy: proportion of indoor feeding mosquitoes

In addition to these, the inputs include **additional bionomics parameters** that either are not currently measured by entomological surveillance, or difficult to measure in the field, therefore restricted information is available. For this reason, they all have the same value in the AnophelesModel database, but the user can change them when data becomes available. These include:

- the relative availability of different non-human hosts, zeta.3, currently not measured by entomological surveillance, but can vary for example in rural versus urban settings. Currently, in absence of better information, we assume that there is no difference in the relative availability of non-human hosts, thus zeta.3 = 1
- the proportion of a day that a mosquito actively seeks a host (td) is set to 0.33 based on data from (2) and (3)
- the time required for a mosquito that has encountered a host to return to host-seeking (tau) is set to 3 days following (4)
- the time (ts) it takes an infected mosquito to become infective (for *Plasmodium* sporozoites to travel to the salivary glands of the mosquito) known to vary between 10-12 days and is set to 10 days following (4)
- oocyst development time (to) usually (3-5 days) is set to 5 days following (5) and (1)

## A.b Defining the host-specific parameters: **def_host_params()**

After defining the mosquito bionomics parameters, the user needs to provide some information about the transition probabilities in absence of interventions, as well as the infectivity of hosts to mosquitoes. These together with the bionomics parameters are used to initialize the model.

The parous rate, $M$, is equal the probability that a mosquito survives an entire feeding cycle, as defined in the model equations from (1):

$$M= P_{f}=\frac{\sum_{i=1}^{n} P_{A^{i}}P_{B^{i}}P_{C^{i}}P_{D^{i}}P_{E^{i}}}{1-P_{A}}$$

where:

n = number of types of hosts (humans and animals)

$P_{A^{i}}$ = probability that a mosquito has found a host of type $i$ and has ended the host seeking stage

$P_{B^{i}}$= probability that a mosquito bites a host of type $i$ after encountering a host of type $i$

$P_{C^{i}}$= probability that a mosquito finds a resting place after biting a host of type $i$

$P_{D^{i}}$= probability that a mosquito survives the resting phase after biting a host of type $i$

$P_{E^{i}}$= probability that a mosquito lays eggs and restarts host-seeking after biting a host of type $i$

$P_{A}$= probability that the mosquito stays in the host seeking stage, equal to ${1-A}_{0}$ where $A_{0}$ is the sac rate, which is one of the input bionomics parameters.

The equation above is used to derive the probabilities $P_{A^{i}}$ in absence of interventions as follows (according to Equations 14 and 15 from the Additional File 2 in (6):

- For human hosts:

$$P_{A^{1}}=\frac{A_{0}M\chi\zeta_{2}P_{B^{2}}P_{C^{2}}}{P_{B^{1}}P_{C^{1}}\left( \left( \chi P_{D^{1}}P_{E^{1}}+(1-\chi)P_{D^{2}}P_{E^{2}} \right)\zeta_{2}P_{B^{2}}P_{C^{2}} \right)}$$

- For animal hosts:

$$P_{A^{2}}=\frac{A_{0}M(1-\chi)}{\left( \chi P_{D^{1}}P_{E^{1}}+(1-\chi)P_{D^{2}}P_{E^{2}} \right)\zeta_{2}P_{B^{2}}P_{C^{2}}}$$

where:

$A_{0}$ = sac rate, an input bionomics parameter

$M$ = parous rate, an input bionomics parameter

$\chi$ = human blood index, an input bionomics parameter

$\zeta_{2}$ = relative availability of animal hosts, equal to 1

$P_{B^{i}}, P_{C^{i}},P_{D^{i}},P_{E^{i}}$ = transition probabilities between the different states of the feeding cycle in absence of interventions (see above), input parameters provided by the user

Therefore, for a solution to exist, the two equations above need to give outputs between 0 and 1, i.e., the survival reflected by the mosquito bionomics parameters (parous rate, sac rate, human blood index) needs to be in agreement with the modelled survival through the transitions probabilities $(P_{B^{i}}, P_{C^{i}},P_{D^{i}},P_{E^{i}})$.

For instances where user-entered values result in this special case, the package gives an erorr: “Simulated mosquito dynamics during the feeding cycle is not reflected by the mosquito bionomics parameters! Consider increasing the values for PBi, PCi, PDi, PEi in the def_host_param() function call.”

Kvi should be set to 0 for animal hosts for *Plasmodium falciparum*. However, this does not generally apply, e.g. for *Plasmodium knowlesi*. Since the package is not exclusively focused on *P. falciparum*, we allow the user to define this value as appropriate for their specific use case.

## A.c Defining activity patterns: **def_activity_patterns()**

Vector and human behaviour is used in the package to calculate the human exposure to mosquitoes and adjust the effects of vector control interventions (see Section C below). Therefore, the user needs to provide information about the biting behaviour of mosquitoes (indoors or outdoors) throughout the day, as well as the location of people within this time (indoors, outdoors, in bed/under a mosquito net). The package comes with a comprehensive database of mosquito and human activity patterns (data object activity_patterns) from which users can choose. Alternatively, users can input their own data.

## A.d Defining intervention effects: **def_intervention_effects()**

Following interventions, mosquitoes die at different stages (each intervention affects mosquitoes at specific stages). The user needs to specify the interventions that they would like to simulate in the model. To do so, they can use existing, published intervention parameterisations available in the interventions_param data object provided with the package, or input new intervention parameterisations. A systematic procedure for this is provided in the package documentation.

# Modelling the effects of vector control interventions

**Table A**: Intervention models included in AnophelesModel. Their specifications as well as references to the publications describing the models and data used are provided. DDT stands for Dichlorodiphenyltrichloroethane.

| **ID in the package** | **Type** | **Active**  **agent/brand** | **Species** | **Duration (years)** | **Resistance** | **Citations** |
| --- | --- | --- | --- | --- | --- | --- |
| IRS01 | IRS | DDT | *An. albimanus* | 0.5 | Yes | (7) |
| IRS10 |  | DDT | *An. gambiae* | 1 | No | (1, 6) |
| IRS12 |  | DDT | *An. gambiae* | 0.5 | No | (6, 8) |
| IRS14 |  | Deltamethrin | *An. gambiae* | 0.5 | No | (6, 9) |
| IRS16 |  | Permethrin | *An. gambiae* | 1 | No | (1, 6) |
| IRS02 |  | Icon 10 CS | *An. gambiae* | 1 | Yes | (10) |
| IRS03 |  | Actellic 300 CS | *An. gambiae* | 1 | Yes | (10) |
| IRS04 |  | Actellic 50 EC | *An. gambiae* | 1 | Yes | (10) |
| IRS05 |  | Bendiocarb | *An. gambiae* | 1 | No | (1, 6) |
| IRS06 |  | Bendiocarb | *An. albimanus* | 0.5 | No | (6, 9) |
| IRS08 |  | Bendiocarb | *An. gambiae* | 0.5 | Yes | (6, 11) |
| IRS17 |  | Lambdacyhalothrin | *An. gambiae* | 0.5 | Yes | (6, 11) |
| LLINs01 | LLINs | PermaNet 2.0 (Akron parameterization) | *An. gambiae* | 3 | Yes | (12, 13) |
| LLINs02 |  | PermaNet 2.0 (Zeneti parameterization) | *An. gambiae* | 3 | No | (12, 13) |
| LLINs03 |  | PermaNet 2.0 (Malanville parameterization) | *An. gambiae* | 3 | No | (12, 13) |
| LLINs04 |  | Lambdacyhalothrin | *An. albimanus* | 3 | No | (12, 13) |
| Screening01 | House screening | - | *An. gambiae* | 3 | - | (1, 6) |

For parameterising the effects of LLIN and IRS interventions, the mosquitoes assessed in experimental hut studies were classified into four categories: UA: unfed alive; UD: unfed dead; FD: fed dead; FA: fed alive (14). These proportions of mosquitoes found in the different categories were used to calculate the probabilities of entering ($P_{ent}$), attacking ($P_{att}$), of dying before feeding ($P_{B\mu}$) and of dying after feeding ($P_{C\mu}$):

$$P_{ent}=\frac{total in treated}{total in control}; P_{att}=\frac{(total -UA)}{total}; P_{B\mu}=\frac{UD}{(UD+FA+FD)};P_{C\mu}=\frac{FD}{(FD+FA)}$$

Where “total in treated” represents the total number of mosquitoes in huts with an intervention, “total in control” represents the total number of mosquitoes in huts without an intervention, and “total” represents the total number of mosquitoes in treated or control huts, being equal to the sum UA + UD + FD + FA.

## B.a LLIN effects models

The data from (15) and the approach described in (16) were used to calculate survival and holed area of a default PermaNet 2.0 net assuming a smooth-compact decay for the survival of the net and a parabolic growth decay for the net holed area.

Each of the probabilities $P_{ent},P_{att}{,P}_{B\mu}$*, or* $P_{C\mu}$ defined before was modelled using a logistic regression approach previously defined in (6) as functions of the holed surface area of the net, *H,* in cm^2^, and the insecticide, *X*, of concentration *[X]* in mg/m^2^:

$$\begin{matrix} \mathrm{logit}\left( P_{ent}\left( X \right) \right)=\beta_{0,ent}+\beta_{1,ent}\gamma\ln\left[ X+1 \right] \\ \mathrm{logit}\left( P_{att}\left( X, H \right) \right)=\beta_{0,att} {+ \beta}_{1,att}\ln\left( H+1 \right)+\beta_{2,att}\gamma\ln\left( \left[ X \right]+1 \right){+ \beta}_{3,att}\gamma\ln\left( \left[ X \right]+1 \right)\ln\left( H+1 \right) \\ \begin{matrix} \mathrm{logit}\left( P_{B\mu}\left( X, H \right) \right)=\beta_{0,B\mu} {+ \beta}_{1,B\mu}\ln\left( H+1 \right)+\beta_{2,B\mu}\gamma\ln\left( \left[ X \right]+1 \right){+ \beta}_{3,B\mu}\gamma\ln\left( \left[ X \right]+1 \right)\ln\left( H+1 \right) \\ \mathrm{logit}\left( P_{C\mu}\left( X, H \right) \right)=\beta_{0,C\mu} {+ \beta}_{1,C\mu}\ln\left( H+1 \right)+\beta_{2,C\mu}\gamma\ln\left( \left[ X \right]+1 \right){+ \beta}_{3,C\mu}\gamma\ln\left( \left[ X \right]+1 \right)\ln\left( H+1 \right) \end{matrix} \end{matrix}$$

where $\beta_{i,att}$, $\beta_{i,B\mu}$, $\beta_{i,C\mu}$ are regression coefficients and $\gamma$ is a scaling factor adjusting for the insecticide concentrations of the different LLIN types against the base parameterization done for PermaNet 2.0.

The deterrency was calculated by comparing the availability of humans using LLINs to mosquitoes, relative to humans that are not protected by LLINs, where $H_{max}$ is the total surface of a net, equal to 192’000 cm^2^, and is substituted for *H* to calculate the values for an unprotected human:

$\frac{\alpha}{\alpha\left( 0 \right)}=\frac{P_{att}\left( X, H \right)P_{ent}\left( X \right)}{P_{att}\left( 0, H_{max} \right)P_{ent}\left( 0 \right)}$ .

A dataset of net durability experiments conducted by the President Malaria Initiative (12) was used to extract decay information about the holed area and survival of 8 net types in 7 countries collected over 8 semesters and to calculate the properties of these types of nets. Spline interpolation was used to estimate the decay of the holed area and of net survival over time. Insecticide decay was assumed to be exponential for all nets. The values for the holed surface area of the net, *H*, and the insecticide concentration *[X]* were estimated for a set of interpolation time points (default is 100 points) across the duration of the intervention (assumed to be 3 years for LLINs) and provided to the series of logistic regression models above to calculate the deterrency, pre- and post-prandial effects.

## B.b IRS effects models

In the AnophelesModel package, IRS is also parameterised in terms of reducing the availability of humans to mosquitoes, and the killing of mosquitoes. Several published parameterisations and models are included (see Table A above for the references to the published models). Accordingly, a subset of IRS parameterizations is specified through their initial effect and assumed type of decay (the decay can be step, Weibull or exponential, cf. models specified in Table A above). Other parameterizations are defined by calculating the values of the effects for 13 estimated, equally-distanced time points across the intervention duration. These were calculated using experimental hut trial data with the formulas for $P_{ent},P_{att}{,P}_{B\mu}$*, or* $P_{C\mu}$ defined above*.* Since some types of IRS kill the mosquitoes resting on walls, several IRS parameterizations treat the effect as a reduction in the probability of surviving the resting phase.

For the IRS parameterisations where Weibull decay is assumed (IRS02-IRS04, cf. Table A), a parameterisation corresponds to a set of initial parameters (repellence, pre-prandial killing effect and post-prandial killing effect), as well as the parameters associated to a Weibull decay (shape and half-life). They were fitted with the following statistical model, for a dataset with $N$ observations indexed by $m$ (for each notation, the superscript “c” indicates the control):

| **Notation** | **Description** |
| --- | --- |
| $t\left( m \right)$ | Time of the observation (in days) |
| $i\left( m \right)$ | Insecticide type of the observation |
| $PB=1-P_{B\mu}$ | Probability of feeding |
| $PC=1-P_{C\mu}$ | Probability of surviving after feeding |
| $F=FA+FD$ | Number of fed mosquitoes |
| $A=UD+FA+FD$ | Number of attacking mosquitoes |
| **Parameters of interest** | |
| $\beta_{i\left( m \right)}$ | Scale parameter of the Weibull distribution for insecticide $i\left( m \right)$ |
| $\kappa_{i\left( m \right)}$ | Shape parameter of the Weibull distribution for insecticide $i\left( m \right)$ |
| $InitialPost{Prandial}_{i\left( m \right)}$ | initial postprandial killing effect for insecticide $i\left( m \right)$ |
| $InitialPr{ePrandial}_{i\left( m \right)}$ | initial preprandial killing effect for insecticide $i\left( m \right)$ |
| $Initial{Repellency}_{i\left( m \right)}$ | initial repellent effect, for insecticide for insecticide $i\left( m \right)$ |

Based on the notations above, for $m\in\left\{ 1:N \right\}:$

$$FA_{m}\sim Binom\left( PC_{m}, F_{m} \right) , with PC_{m}=PC_{t\left( m \right)}^{c}\left( 1-InitialPos{tPrandial}_{i(m)}exp(-{\beta_{i\left( m \right)}t(m)}^{\kappa_{i\left( m \right)}}) \right)$$

$$F_{m}\sim Binom\left( PB_{m}, A_{m} \right), with PB_{m}=PB_{t\left( m \right)}^{c}\left( 1-InitialPr{ePrandial}_{i\left( m \right)} exp(-{\beta_{i\left( m \right)}t(m)}^{\kappa_{i\left( m \right)}}) \right)$$

$$A_{m}\sim Binom\left( P{Att}_{m}, T_{m} \right), with P{Att}_{m}=P{Att}_{t\left( m \right)}^{c}\left( 1-InitialR{epellency}_{i\left( m \right)} exp(-{\beta_{i\left( m \right)}t(m)}^{\kappa_{i\left( m \right)}}) \right)$$

With random effects per day defined as:

$$logit\left( PC_{t\left( m \right)}^{c} \right)\sim N\left( \mu_{t\left( m \right)}^{C}, 1/\sigma_{t\left( m \right)}^{C} \right)$$

$$logit\left( PB_{t\left( m \right)}^{c} \right)\sim N\left( \mu_{t\left( m \right)}^{B}, {1/\sigma}_{t\left( m \right)}^{B} \right)$$

$$logit\left( P{Att}_{t\left( m \right)}^{c} \right)\sim N(\mu_{t\left( m \right)}^{A}, {1/\sigma}_{t\left( m \right)}^{A})$$

## B.c Combinations of interventions

For an intervention $i$, deployed at time $t=0$ and at coverage $C_{i}$, the intervention effects at subsequent times $t>0$ for protected humans depend on the values of the transition probabilities $\alpha_{i}\left( t \right), P_{B_{i}}\left( t \right), P_{C_{i}}\left( t \right), P_{D_{i}}\left( t \right), P_{E_{i}}\left( t \right)$, which may change over time, (because of acquisition of holes in nets, or decay of insecticidal effect). The values of these probabilities for non-intervened humans (not protected by the intervention), are $\alpha_{u}\left( t \right), P_{B_{u}}\left( t \right), P_{C_{u}}\left( t \right), P_{D_{u}}\left( t \right), P_{E_{u}}\left( t \right)$. It follows that ratios of the intervened to non-intervened effects are:

$$R_{\alpha_{i}}\left( t \right)= \alpha_{i}(t)/\alpha_{u}\left( t \right)$$

$$R_{B_{i}}\left( t \right)= P_{B_{i}}(t)/P_{B_{u}}\left( t \right)$$

$$R_{C_{i}}\left( t \right)= P_{C_{i}}(t)/P_{C_{u}}\left( t \right)$$

$$R_{D_{i}}\left( t \right)= P_{D_{i}}(t)/P_{D_{u}}\left( t \right)$$

$$R_{E_{i}}\left( t \right)= P_{E_{i}}(t)/P_{E_{u}}\left( t \right)$$

The effective coverage at time $t$ is then $C_{i}^{*}\left( t \right)= C_{i}S_{i}(t)$, where $S_{i}\left( 0 \right)=1$, and $S_{i}\left( t \right)$ is the survival of the intervention (measuring attrition, in the case of LLINs). The impact on transmission at time $t$ is obtained from the mosquito feeding cycle model with the categories intervened and non-intervened human hosts in the ratio $C_{i}^{*}\left( t \right):1-C_{i}^{*}\left( t \right).$

Where there is a second intervention, $j$, deployed at coverage $C_{j}$, and the corresponding intervention dependent transition probabilities $\alpha_{j}\left( t \right), P_{B_{j}}\left( t \right), P_{C_{j}}\left( t \right), P_{D_{j}}\left( t \right), P_{E_{j}}\left( t \right)$, the values applicable to the individuals that are protected by both interventions are obtained by treating the two interventions as having independent efficacies for each stage in the cycle so that:

$$\alpha_{ij}\left( t \right)= \alpha_{u}(t)R_{\alpha i}\left( t \right)R_{\alpha j}\left( t \right)$$

$$P_{B_{ij}}\left( t \right)= P_{B_{u}}(t)R_{B_{i}}\left( t \right)R_{B_{j}}\left( t \right)$$

$$P_{C_{ij}}\left( t \right)= P_{C_{u}}(t)R_{C_{i}}\left( t \right)R_{C_{j}}\left( t \right)$$

$$P_{D_{ij}}\left( t \right)= P_{D_{u}}(t)R_{D_{i}}\left( t \right)R_{D_{j}}\left( t \right)$$

$$P_{E_{ij}}\left( t \right)= P_{E_{u}}(t)R_{E_{i}}\left( t \right)R_{E_{j}}\left( t \right)$$

Let $C_{ij}$ be the proportion of the population who receive both interventions at $t=0$. The coverage vector for all four possible categories of human hosts (non-intervened, protected by intervention $i$, protected by intervention $j$, protected by both interventions is then:

$$C=\left( \begin{matrix} 1-C_{i}-C_{j}+C_{ij} \\ C_{i}-C_{ij} \\ \begin{matrix} C_{j}-C_{ij} \\ C_{ij} \end{matrix} \end{matrix} \right)$$

Assuming the survival of the two interventions to be independent, the proportion of the population effectively covered by both interventions at time $t$ is:

$$C_{i}^{*}\left( t \right)= C_{ij}S_{i}(t) S_{j}(t)$$

and the effective coverage vector for the four categories of hosts at time $t$ is:

$$C^{*}=\left( \begin{matrix} C^{*}[1] \\ C^{*}[2] \\ \begin{matrix} C^{*}[3] \\ C^{*}[4] \end{matrix} \end{matrix} \right)=\left( \begin{matrix} 1-C_{i}^{*}\left( t \right)-C_{j}^{*}\left( t \right)+C_{ij}^{*}\left( t \right) \\ C_{i}^{*}\left( t \right)-C_{ij}^{*}\left( t \right) \\ \begin{matrix} C_{j}^{*}\left( t \right)-C_{ij}^{*}\left( t \right) \\ C_{ij}^{*}\left( t \right) \end{matrix} \end{matrix} \right).$$

The AnophelesModel package estimates impact for two categories of human hosts: intervened and not-intervened. Therefore, the parameter values for the intervened category are weighted averages of the values for the four possible categories, where the weighting captures the different proportions of mosquitoes reaching the corresponding stage of the cycle (and hence the proportion of mosquitoes exposed to each intervention combination at each stage). These weighted parameter estimates are defined recursively as follows:

$$\alpha_{w}\left( t \right)={C^{*}\left[ 1 \right]\alpha_{u}\left( t \right)+ C}^{*}\left[ 2 \right]\alpha_{i}\left( t \right)+ C^{*}\left[ 3 \right]\alpha_{j}\left( t \right)+C^{*}[4]\alpha_{ij}\left( t \right)$$

$$P_{B_{w}}\left( t \right)=\frac{{C^{*}\left[ 1 \right]\alpha_{u}\left( t \right)P_{B_{u}}\left( t \right)+ C}^{*}\left[ 2 \right]\alpha_{i}\left( t \right)P_{B_{i}}(t)+ C^{*}\left[ 3 \right]\alpha_{j}\left( t \right)P_{B_{j}}(t)+C^{*}\left[ 4 \right]\alpha_{ij}\left( t \right)P_{B_{ij}}(t)}{\alpha_{w}\left( t \right)}$$

$$P_{C_{w}}\left( t \right)=\frac{{C^{*}\left[ 1 \right]\alpha_{u}\left( t \right)P_{B_{u}}\left( t \right)P_{C_{u}}\left( t \right)+C}^{*}\left[ 2 \right]\alpha_{i}\left( t \right)P_{B_{i}}\left( t \right)P_{C_{i}}\left( t \right)}{\alpha_{w}\left( t \right)P_{B_{w}}\left( t \right)}$$

$$+ \frac{C^{*}\left[ 3 \right]\alpha_{j}\left( t \right)P_{B_{j}}(t)P_{C_{j}}(t)+C^{*}\left[ 4 \right]\alpha_{ij}\left( t \right)P_{B_{ij}}(t)P_{C_{ij}}(t)}{\alpha_{w}\left( t \right)P_{B_{w}}\left( t \right)}$$

$$P_{D_{w}}\left( t \right)=\frac{{C^{*}\left[ 1 \right]\alpha_{u}\left( t \right)P_{B_{u}}\left( t \right)P_{C_{u}}\left( t \right)P_{D_{u}}\left( t \right)+C}^{*}\left[ 2 \right]\alpha_{i}\left( t \right)P_{B_{i}}(t)P_{C_{i}}(t)P_{D_{i}}(t)}{\alpha_{w}\left( t \right)P_{B_{w}}\left( t \right)P_{C_{w}}\left( t \right)}+ \frac{C^{*}\left[ 3 \right]\alpha_{j}\left( t \right)P_{B_{j}}(t)P_{C_{j}}(t)P_{D_{i}}(t)+C^{*}\left[ 4 \right]\alpha_{ij}\left( t \right)P_{B_{ij}}(t)P_{C_{ij}}(t)P_{D_{ij}}(t)}{\alpha_{w}\left( t \right)P_{B_{w}}\left( t \right)P_{C_{w}}\left( t \right)}$$

$$P_{E_{w}}\left( t \right)==\frac{{C^{*}\left[ 1 \right]\alpha_{u}\left( t \right)P_{B_{u}}\left( t \right)P_{C_{u}}\left( t \right)P_{D_{u}}\left( t \right)P_{E_{u}}\left( t \right)+ C}^{*}\left[ 2 \right]\alpha_{i}\left( t \right)P_{B_{i}}(t)P_{C_{i}}(t)P_{D_{i}}(t)P_{E_{i}}(t)}{\alpha_{w}\left( t \right)P_{B_{w}}\left( t \right)P_{C_{w}}\left( t \right)P_{D_{w}}\left( t \right)}+ \frac{\left[ 3 \right]\alpha_{j}\left( t \right)P_{B_{j}}(t)P_{C_{j}}(t)P_{D_{j}}(t)P_{E_{j}}(t)+C^{*}\left[ 4 \right]\alpha_{ij}\left( t \right)P_{B_{ij}}(t)P_{C_{ij}}(t)P_{D_{ij}}(t)P_{E_{ij}}(t)}{\alpha_{w}\left( t \right)P_{B_{w}}\left( t \right)P_{C_{w}}\left( t \right)P_{D_{w}}\left( t \right)}$$

# Calculating human exposure to mosquitoes

The mosquito and human activity patterns included in the package and used to calculate human exposure to mosquitoes consist of time series measurements taken at consecutive time points throughout the day, namely:

- Indoor biting rate (proportion of overall human biting happening indoors at time t): $b_{t}^{indoors}$, with $1\leq t\leq n$
- Outdoor biting rate (proportion of overall human biting happening outdoors at time t): $b_{t}^{outdoors}$, with $1\leq t\leq n$
- Proportion of humans located indoors at time t: $h_{t}^{indoors}$, with $1\leq t\leq n$
- Proportion of humans located in bed at time t: $h_{t}^{bed}$, with $1\leq t\leq n$

The proportion of humans located outdoors at time t, $h_{t}^{outdoors}$, with $1\leq t\leq n$ is calculated from the proportion of humans located indoors: $h_{t}^{outdoors}= {1- h}_{t}^{indoors}$.

Within the package, the proportions of human biting indoors and outdoors are normalized and coupled by the means of the endophagy, denoted with Ɛ in the following, to estimate the biting rates:

$$B_{t}^{indoors}= \frac{b_{t}^{indoors}}{\sum_{t=1}^{n} b_{t}^{indoors}}\varepsilon$$

$B_{t}^{outdoors}= \frac{b_{t}^{outdoors}}{\sum_{t=1}^{n} b_{t}^{outdoors}} (1- \varepsilon)$.

Previous studies have used mosquito feeding behavior data collected through light traps or human landing catches (HLC) to estimate the proportion of bites that humans receive from mosquitoes indoors and outdoors and to directly affect effectiveness of vector control interventions (17). Coupled with human behavior data, these proportions have been adjusted in further studies to estimate behavior-adjusted exposure of humans to mosquito bites (18). However, this mosquito biting data does not include information about the amount of biting happening outside the measured hours (usually between 6pm and 6am and not for the 24-hour period). To correct this, in the AnophelesModel package, the indoor and outdoor biting rates are further adjusted according to the mosquito endophagy. Precisely, without the coupling, there is no relative link between the estimated indoor and outdoor biting rates as no information is contained in the measurements about the proportion of each type of biting out of the total biting happening during the day. The overall reduction in total biting (indoor and outdoor) is important for estimating the impact of interventions which target only one type of biting, such as LLINs. By adjusting these rates, we can calculate the different types of human exposure to mosquitoes (indoor, in bed, outdoor exposure) relative to the total exposure of humans to mosquitoes and use them to adjust the effects of vector control interventions accordingly.

The total human exposure to mosquitoes is then calculated as follows:

$E^{total}=\sum_{t=1}^{n} \left( B_{t}^{indoors} h_{t}^{indoors} + B_{t}^{outdoors}h_{t}^{outdoors} \right)$.

In most cases, humans are indoors while sleeping ($h_{t}^{indoors}\geq h_{t}^{bed}$). For cases where $h_{t}^{indoors}<h_{t}^{bed}$, we assign the difference to outdoor sleeping. The indoor and outdoor exposures of humans to mosquitoes while in bed are:

$E^{indoor|bed}=\frac{\sum_{t=1}^{n} B_{t}^{indoors}{min(h}_{t}^{indoors},h_{t}^{bed})}{E^{total}}$ , and

$E^{outdoor|bed}=\frac{\sum_{t=1}^{n} B_{t}^{outdoors}{max(h_{t}^{bed}-h}_{t}^{indoors}, 0)}{E^{total}}$ , respectively.

Finally, the total indoor and outdoor exposures of humans to mosquitoes are:

$E^{indoor}= \frac{\sum_{t=1}^{n} B_{t}^{indoors} h_{t}^{indoors}}{E^{total}}$, and

$E^{outdoor}= \frac{\sum_{t=1}^{n} B_{t}^{outdoors} h_{t}^{outdoors}}{E^{total}}$, respectively.

Once the different types of exposures of humans to mosquitoes have been calculated, the deterrency, pre-prandial and post-prandial effects of the interventions are adjusted by multiplying them by the relevant exposure coefficient (summarized in Table B below).

***Table B****: Exposure multipliers used to adjust the effects of vector control interventions. These effects act on the deterrency (*α_i_), preprandial effect (represented through the transition probability P_Bi_ of successfully completing the host seeking stage) and postprandial effect (represented through either the probability P_Ci_ that a mosquito has successfully fed or the probability P_Di_ that a mosquito has successfully survived the resting phase). φ represents the mosquito endophily.

|  |  | **Parameters (transition probabilities) within the model affected by interventions** | | | |
| --- | --- | --- | --- | --- | --- |
|  |  | $\boldsymbol{\alpha}_{\boldsymbol{i}}$ | $\boldsymbol{P}_{\boldsymbol{Bi}}$ | $\boldsymbol{P}_{\boldsymbol{Ci}}$ | $\boldsymbol{P}_{\boldsymbol{Di}}$ |
| **Interventions** | LLINs | $E^{indoor\vert bed}$ | $E^{indoor\vert bed}$ | $E^{indoor\vert bed}$ | $NA$ |
|  | IRS, House screening | $E^{indoor}$ | $E^{indoor}$ | $E^{indoor}$ | $\varphi$ |

# Incorporating parameter uncertainty

Due to its lightweight implementation, AnophelesModel is fast and can be used to estimate intervention impact for a large set of input parameter combinations. The user can thus easily investigate how the model outputs vary depending on the input parameters, propagate parameter uncertainty, and perform sensitivity analysis. To illustrate this, the package contains an in built function, *calculate_impact_var()*, which performs sampling from the confidence intervals of the mosquito bionomics parameters included in the package database and estimates the resulting variation in vectorial capacity. Similarly, for any input data, the user can build an extensive set of parameter combinations and use AnophelesModel to assess the resulting variations in the estimated vectorial capacity.

# Considering multiple mosquito species

The package allows estimating the effects of vector control interventions on the vectorial capacity for a mosquito species at a time. Nevertheless, it is possible to calculate the combined reduction in vectorial capacity for several mosquito species through a weighted average by using as weights their relative density in a geographical setting. The composite vectorial capacity $V$ for $n$ mosquito species would be:

$$V= \sum_{i=1}^{n} V_{i}d_{i}$$

with $d_{i}$ representing the proportion of mosquito species $i$ out of the species composition of a given geographical setting, with $\sum_{i=1}^{n} d_{i}=1$, and $V_{i}$ representing the vectorial capacity associated with mosquito species $i$. Relative densities of various mosquito species can be estimated from field studies or from publicly available datasets with global estimates such as provided in (19).

# Using AnophelesModel to parameterize OpenMalaria

The AnophelesModel package can be used to generate specifications for vectors and vector control interventions in the OpenMalaria model. OpenMalaria requires a configuration file in XML format which includes all the specifications of a simulation. The objects required for modelling vector characteristics and the effects of vector control interventions in OpenMalaria are XML snippets for inclusion in the scenario XML. Entomological characteristics are defined through an entomology XML snippet, and intervention effects can be defined through the “generic vector intervention” (GVI) XML snippet (further information about OpenMalaria XML definitions is provided at <https://github.com/SwissTPH/openmalaria/wiki>). The GVI snippet includes the definition of decay and initial effect parameters for deterrency, pre- and post-prandial killing effects of interventions. In OpenMalaria, the intervention effects modelled through GVI components can be associated with one of seven possible decay functions. AnophelesModel uses nonlinear least squares (R package minpack.lm version 1.2-2) to fit in turn each of the seven decay functions to the time series of estimated intervention effects and chooses the decay with the best fit (smallest residual sum of squares). The XML components needed for OpenMalaria simulation specifications can then be generated with the package.

For illustrating using OpenMalaria the example considering the Kenyan-like and PNG-like settings described in the main manuscript, we informed the model parameters regarding seasonality of transmission, entomological, and vector control interventions with geographic-specific values. To do so, we estimated the geographic-specific entomological parameters (Fig 2A from the main manuscript) and intervention effects decays (Fig 3A from the main manuscript) of LLINs deployment using AnophelesModel and further incorporated them in OpenMalaria simulations of malaria dynamics. OpenMalaria version 44 was used for this analysis. Populations of 10,000 people in each setting were simulated starting January 1999, with a single LLINs deployment in January 2023 at 60% coverage. Case management was the only other intervention present in the simulation, deployed from the beginning, and was set to 50% effective coverage for both settings. Coverage of an intervention was defined as the proportion of people protected against malaria infection by that intervention.

For simplicity, in this simulation example, malaria transmission was treated as proportional to monthly rainfall, an assumption that is not implicit in real-world settings. Rainfall data was extracted from WorldClim (59) and shifted by a lag period of 30 days to consider the delay in mosquito density, emergence and infection. The Kenyan-like simulation used the rainfall profile of the Kisumu region, and the PNG-like simulation that of the Momase region. For the sake of comparison, the transmission intensity prior to start of the interventions deployment was considered the same in both settings by choosing an initial annual entomological inoculation rate of 15 infective bites per person per year for both simulated settings.

*Plasmodium falciparum* prevalence in all ages over time was simulated for the two settings (Fig A below). As expected, the impact in reducing prevalence by LLINs deployment was lower in the PNG-like setting compared to the Kenya-like setting. By allowing accurate incorporation of intervention effects in models of malaria transmission such as OpenMalaria, AnophelesModel facilitates exploring further, more complex intervention scenarios, such as combining vector control with drug interventions or supplementing the LLINs deployments with other interventions potentially targeting outdoor biting in PNG.

**
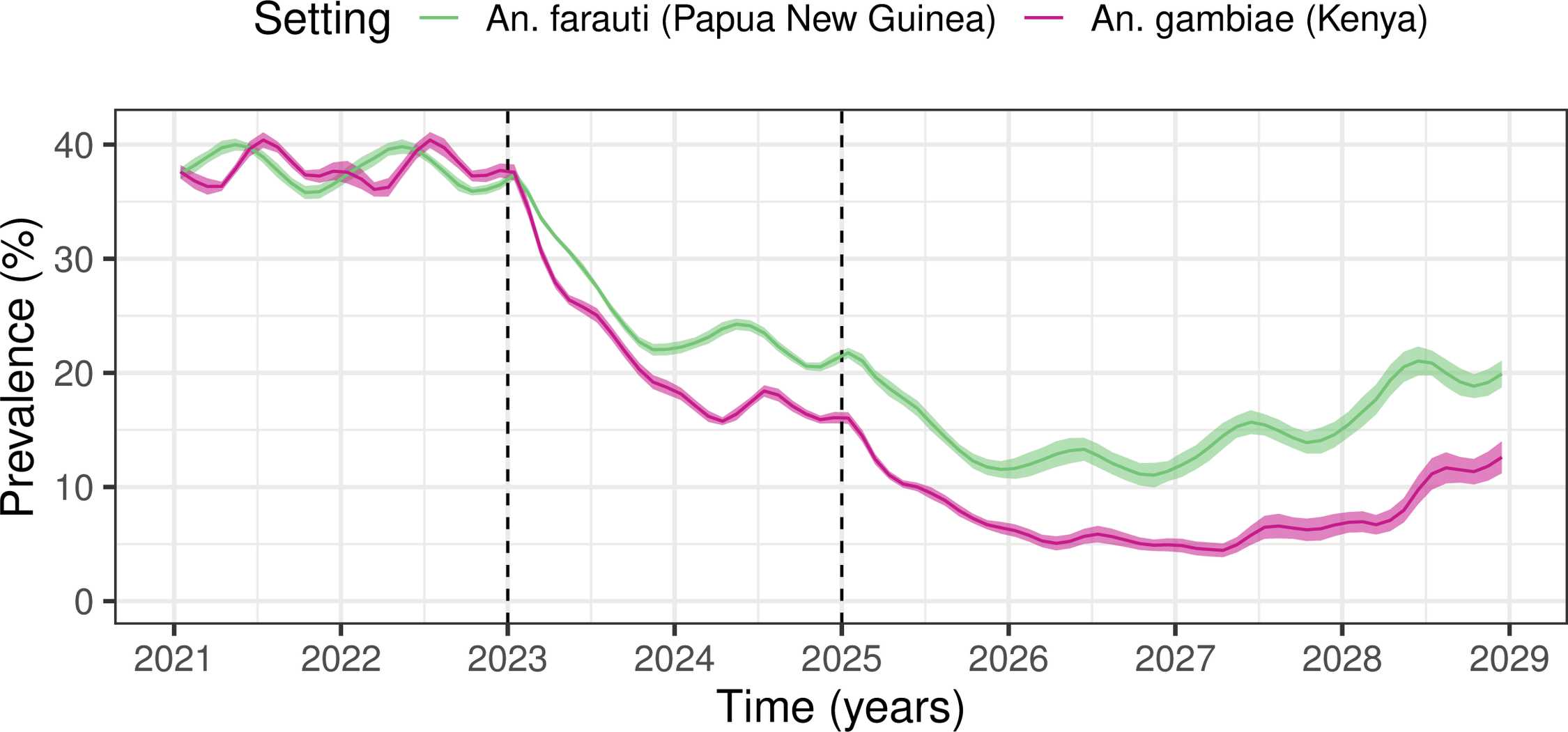
**

**Fig A: Simulation of the impact of LLINs deployment in OpenMalaria.** XML snippets produced by AnophelesModel were used in OpenMalaria to model the entomology and effects of LLINs deployments in Kenyan-like and PNG-like settings and to simulate all-age prevalence. Two deployments of LLINs were simulated in January 2023 and 2025 (dashed lines). The prevalence during the simulated years (2021-2029) is displayed for both settings. The shaded areas around the curve show the uncertainty in the estimated prevalence.

**References**

1. Chitnis N, Smith T, Steketee R. A mathematical model for the dynamics of malaria in mosquitoes feeding on a heterogeneous host population. Journal of Biological Dynamics. 2008;2(3):259-85.

2. Saul A. Zooprophylaxis or zoopotentiation: the outcome of introducing animals on vector transmission is highly dependent on the mosquito mortality while searching. Malaria journal. 2003;2:1-18.

3. Killeen GF, Kihonda J, Lyimo E, Oketch FR, Kotas ME, Mathenge E, et al. Quantifying behavioural interactions between humans and mosquitoes: evaluating the protective efficacy of insecticidal nets against malaria transmission in rural Tanzania. BMC infectious diseases. 2006;6:1-10.

4. Buxton P, Leeson H. Anopheline mosquitoes: Life history. Malariology, a comprehensive survey of all aspects of this group of diseases from a global standpoint. 1949:257-83.

5. Service M. Mosquito ecology: field sampling methods. 1976.

6. Briët OJ, Impoinvil DE, Chitnis N, Pothin E, Lemoine JF, Frederic J, et al. Models of effectiveness of interventions against malaria transmitted by Anopheles albimanus. Malaria journal. 2019;18(1):1-12.

7. Bangs MJ. The susceptibility and behavioral response of Anopheles albimanus Weidemann and Anopheles vestitipennis Dyar and Knap (Diptera: Culicidae) to insecticides in northern Belize, Central America: Uniformed Services University of the Health Sciences; 1999.

8. Kuhlow F. Field experiments on the behaviour of malaria vectors in an unsprayed hut and in a hut sprayed with DDT in Northern Nigeria. Bulletin of the World Health Organization. 1962;26(1):93.

9. Bown DN, Rodríguez M, Arredondo-Jimenez JI, Loyola E, Rodriguez MdC. Age structure and abundance levels in the entomological evaluation of an insecticide used in the control of Anopheles albimanus in southern Mexico. J Am Mosq Control Assoc. 1991;7(2):180-7.

10. Tchicaya ES, Nsanzabana C, Smith TA, Donzé J, de Hipsl ML, Tano Y, et al. Micro-encapsulated pirimiphos-methyl shows high insecticidal efficacy and long residual activity against pyrethroid-resistant malaria vectors in central Côte d’Ivoire. Malaria journal. 2014;13:1-13.

11. Agossa FR, Aïkpon R, Azondékon R, Govoetchan R, Padonou GG, Oussou O, et al. Efficacy of various insecticides recommended for indoor residual spraying: pirimiphos methyl, potential alternative to bendiocarb for pyrethroid resistance management in Benin, West Africa. Transactions of the Royal Society of Tropical Medicine and Hygiene. 2014;108(2):84-91.

12. Briet O, Koenker H, Norris L, Wiegand R, Vanden Eng J, Thackeray A, et al. Attrition, physical integrity and insecticidal activity of long-lasting insecticidal nets in sub-Saharan Africa and modelling of their impact on vectorial capacity. Malaria journal. 2020;19(1):1-15.

13. Randriamaherijaona S, Briët OJ, Boyer S, Bouraima A, N’Guessan R, Rogier C, et al. Do holes in long-lasting insecticidal nets compromise their efficacy against pyrethroid resistant Anopheles gambiae and Culex quinquefasciatus? Results from a release–recapture study in experimental huts. Malaria journal. 2015;14(1):1-22.

14. Briët OJ, Hardy D, Smith TA. Importance of factors determining the effective lifetime of a mass, long-lasting, insecticidal net distribution: a sensitivity analysis. Malaria journal. 2012;11(1):1-27.

15. Morgan J, Abilio AP, do Rosario Pondja M, Marrenjo D, Luciano J, Fernandes G, et al. Physical durability of two types of long-lasting insecticidal nets (LLINs) three years after a mass LLIN distribution campaign in Mozambique, 2008-2011. Am J Trop Med Hyg. 2015;92(2):286-93.

16. Briët OJ, Penny MA. Repeated mass distributions and continuous distribution of long-lasting insecticidal nets: modelling sustainability of health benefits from mosquito nets, depending on case management. Malaria journal. 2013;12(1):1-19.

17. Sherrard-Smith E, Skarp JE, Beale AD, Fornadel C, Norris LC, Moore SJ, et al. Mosquito feeding behavior and how it influences residual malaria transmission across Africa. Proceedings of the National Academy of Sciences. 2019;116(30):15086-95.

18. Monroe A, Moore S, Okumu F, Kiware S, Lobo NF, Koenker H, et al. Methods and indicators for measuring patterns of human exposure to malaria vectors. Malaria journal. 2020;19(1):1-14.

19. Sinka ME. Global distribution of the dominant vector species of malaria. Anopheles mosquitoes-New insights into malaria vectors: IntechOpen; 2013.
