# Supplementary material for: AnophelesModel: An R package to interface mosquito bionomics, human exposure and intervention effects with models of malaria intervention impact
Source: PLoS Comput Biol. 2024 Sep 13;20(9):e1011609. doi: 10.1371/journal.pcbi.1011609 (PMC11424000; doi:10.1371/journal.pcbi.1011609)
Supplement: Attachment — Submitted filename: comments.docx [file pcbi.1011609.s002.docx]

I can see the potential of this contribution to the field that may prove a regularly used package by researchers, programmers and decision makers. The manuscript is clear and well written, the Figures are informative and it fits well with the journal’s scope. The package worked generally well when using default parameters, however when using the custom settings (particularly for probabilities flowing from host seeking to oviposition), the example custom estimates did not work and neither did a range of other probabilities (see minor comments). It would be useful to determine the range of probabilities that do work so that the user selection could be restricted within this – especially wherever data are available to support the choice (for example, falciparum, to my knowledge only infects humans so Kvi: Proportion of susceptible mosquitoes that become infected after biting, for animals, has to take the value 0 and the user should be restricted to choosing this estimate). The results that could be produced in the absence of this guidance are a little concerning, so I think putting in these restrictions, potentially offering default ranges for mosquitoes other than gambiae for def_host_params() would be very useful indeed.

I have one other major concern for the authors to consider and otherwise very minor comments and suggestions for future directions.

The **major concern** is the presentation of impacts from interventions without any apparent validation. The uncertainty shown is minimal. I think it could be hugely useful to

i) Include some validations: to do this, you could use data from PMI, or RCTs that tracked mosquitoes over time in a baseline year and then into the intervention year. Plotting the cross sectional estimates for reduction in vectorial capacity onto the outputs could inform whether it is sensible to show more uncertainty in the figures, and reassure the user on inferring from these analyses
ii) Include explicit warnings that these results are not validated and are dependent on the assumptions of the underlying mechanisms.

With this in mind, I am not sure it is sensible to include house screening immediately, unless you can validate the estimated impacts. In my experience, screening of houses can lead to very variable outcomes depending on the house style, the degree of screening, the type of materials used for netting, the inclusion or not of insecticide on this material etc… so to show simply a linear reduction in vectorial capacity may not be too useful and may detract confidence in using the otherwise helpful package.

There is good flexibility in the package and the instructions are very helpful and comprehensive (with the already mentioned concerns over ranges for probabilities that could be restricted).

**Minor comments**

The online info needs updating for the now published Lemant et al, - currently referenced as “in preparation”: [AnophelesModel Documentation • AnophelesModel (swisstph.github.io)](https://swisstph.github.io/AnophelesModel/articles/AnophelesModel.html#introduction) in the Mosquito bionomics section of the database explanation

vec_ent_param

There is Anopheles gambiae and Gambiae

There is Anopheles funestus and funestus

Presumably these are the same species (complex?) – when the Bayes fitting was performed, were these grouped? The values for these are quite different (e.g. see Chi)

As Zeta (relative availability of different non-humans hosts) is 1 for the full data base is this indicative of a parameter you would like to estimate in the future but currently cannot gain sufficient data for? Or does 1 indicate something specific?

The activity patterns data base is hugely useful and disaggregated. Thank you

Running through the help documentation, the function

plot_impact_species(impacts, "VC_red")

worked only when probabilities were high enough when using

model_params = build_model_obj(custom_ent_params, custom_host_params, custom_activity_obj, host_pop)

From the instructions:

For using a different, custom parameterisation, first we need to build a data frame with the same structure as host_ent_params:

species_name = "Anopheles example"

host = [c](https://rdrr.io/r/base/c.html)("human", "animal")

PBi = [c](https://rdrr.io/r/base/c.html)(0.67, 0.44)

PCi = [c](https://rdrr.io/r/base/c.html)(0.33, 0.434)

PDi = [c](https://rdrr.io/r/base/c.html)(0.647, 0.344)

PEi = [c](https://rdrr.io/r/base/c.html)(0.672, 0.214)

Kvi = [c](https://rdrr.io/r/base/c.html)(0.52, 0.314)

custom_params_tab = [cbind.data.frame](https://rdrr.io/r/base/cbind.html)(species_name, host, PBi, PCi, PDi, PEi, Kvi)

Afterwards, we can provide this data frame to the relevant function for defining the host parameters:

```
custom_host_params = def_host_params(mosquito_species = species_name, host_table = custom_params_tab)
#> [1] "Using host-specific entomological parameters provided by the user."
```

Using the following: model_params = build_model_obj(custom_ent_params, default_host_params, custom_activity_obj, host_pop)

Worked as per the example.

I tried various combinations of the probabilities, many gave very odd outputs in the plot

p = [plot_impact_species](https://swisstph.github.io/AnophelesModel/reference/plot_impact_species.html)(impacts, "VC_red")

[plot](https://rdrr.io/r/graphics/plot.default.html)(p)

Generally, as long as the human probabilities are high enough, there is a reasonable return. It would be sensible to explore the range in the probabilities that give reasonable solutions and restrict users to selecting from these ranges. Or, set assumed probabilities for major vectors to guide the user. As noted Kvi for animals should surely be 0 for *falciparum*. Should input probabilities here always be assumed in the absence of interventions?

Why can the user not alter PAi?

Perhaps add in a Box to the main manuscript to define the main aspects of mosquito life cycles for the reader (human blood index, sac rate etc…)

Once the above are considered, I support the publication of this manuscript and look forward to the community making use of the database and informative functions that the team have put together.

Adding in other interventions as next steps makes good sense. Providing some validations to the outputs would be highly worth the effort, and would add support for users, particularly those wanting to make use of the tool to support decisions on procurement and deployment of various intervention options. Guiding the user by placing some data-informed restrictions on input values would be sensible. Another useful step would be to enable the combination of interventions to be explored. In addition, providing some guidance on how you would suggest multiple species to be considered. Perhaps you could look at weighted averages for estimates that represent species composition. Again, with all these steps validation would prove a worthy exercise.
