# Supplementary material for: AnophelesModel: An R package to interface mosquito bionomics, human exposure and intervention effects with models of malaria intervention impact
Source: PLoS Comput Biol. 2024 Sep 13;20(9):e1011609. doi: 10.1371/journal.pcbi.1011609 (PMC11424000; doi:10.1371/journal.pcbi.1011609)
Supplement: Attachment — Submitted filename: PCOMPBIOL review.docx [file pcbi.1011609.s003.docx]

This package seems like a valuable contribution to the field, providing accessible databases of important mosquito, human, and intervention parameters, and integrating them into a user-friendly package to estimate the location-specific impacts of mosquito-borne disease intervention strategies. The package documentation linked in the article is easy to follow and thorough, and the article submitted here provides a helpful overview of the package functionality along with a specific use-case example. I believe this publication would be a valuable contribution to the field, made stronger by a few simple changes to help potential users understand what data they provide and how it will be utilized. These changes are detailed below.

**Content Comments:**

Abstract lines 23-25 do not give me a clear understanding of what this package does. Borrowing the phrasing from your GitHub documentation, perhaps you could rephrase this to: “AnophelesModel is an online, open-access, R package that quantifies the impact of vector control interventions using mosquito species and location-specific characteristics.”

Figure 1: I do not follow the model of mosquito feeding cycle and could use a description of what states are included (A-E) in the caption, or perhaps more clarity in the figure. I see 5 state categories (A-E) but only four category descriptions (host seeking, feeding, resting, ovipositing). The fifth state of “searching for a resting place” is specified in line 106 but should be included here for clarity.

Figure 1: What are the different colored lines in the “Reduction in vectorial capacity” subfigure? If they are meant to represent two different control measures, perhaps label them as such.

Design and Implementation section (line 91): Specify exactly what data the user needs to input for a model simulation.

Line 95: Define vectorial capacity here instead of on line 110.

Line 109-111: It would be helpful to understand how intervention effects reduce model transition probabilities. Do mosquitoes just stay in each state for longer due to interventions? Or does it reduce the number of mosquitoes the model is initialized with?

Line 125: My interpretation of what follows this sentence is that interventions may deter mosquito-human interaction (skipping feeding state and potentially increasing death rate) or invoke mosquito death at some rate either prior to or after feeding. These impacts cannot be expressed as simple reductions in the rate parameters of Figure 1, middle panel, and I think the figure would be more helpful if the actual intervention effects were better captured! Extending your model diagram from <https://swisstph.github.io/AnophelesModel/articles/AnophelesModel.html> with arrows to indicate the impacts of intervention measures would be helpful for understanding.

Figure 2: The first caption sentence was not clear to me. Perhaps rephrase to “The package provides (A) entomological parameters, (B) mosquito biting patterns, (C) human activity patterns, and (D) intervention properties, which can be used to…”

Figure 2: I do not see the gray area mentioned for panel (B).

Figure 2: Add a description for panel C as included for all other subplots.

Figure 2D: What is a “semester” on the horizontal axis? Perhaps include this in the caption.

Figure 4: While I know this example is mostly included to showcase how model functionality and the ease of pipelining to OpenMalaria, it may be more interesting to show an example where the two treatment strategies result in drastically different impacts. This could likely be achieve by simply simulating multiple treatment rounds in OpenMalaria.

**Grammar Comments:**

Line 23: Remove comma after “open-access”

Line 27: remove word “on”

Line 164: “using” -> “use of”

Line 168: “are adjusted by multiplying them by the…” -> “are scaled by the…”

Line 177 and 184: Add comma before “and” in compound sentence.

Line 189: Add “with” after “associated”.
