# Supplementary material for: AnophelesModel: An R package to interface mosquito bionomics, human exposure and intervention effects with models of malaria intervention impact
Source: PLoS Comput Biol. 2024 Sep 13;20(9):e1011609. doi: 10.1371/journal.pcbi.1011609 (PMC11424000; doi:10.1371/journal.pcbi.1011609)
Supplement: Attachment — Submitted filename: Golumbeanu_PlosCB_ResponseToReviews.docx [file pcbi.1011609.s004.docx]

Response to Editorial Staff and Reviewers for:

AnophelesModel: An R package to interface mosquito bionomics, human exposure and intervention effects with models of malaria intervention impact

Monica Golumbeanu, Olivier Briët, Clara Champagne, Jeanne Lemant, Munir Winkel, Barnabas Zogo, Maximilian Gerhards, Marianne Sinka, Nakul Chitnis, Melissa Penny, Emilie Pothin, Tom Smith

**Editor's synthesis**: all three reviewers were positive and recommended minor revisions. However, I've chosen to mark this as "major revisions" simply because the three sets of recommendations are generally complementary to each other.

We thank the Editor and the three Reviewers for considering our manuscript. We highly appreciate their useful and constructive comments, which helped strengthen our manuscript about the AnophelesModel package and analysis. In the following, we address all the reviewer comments (in black and marked with a highlighted number) and specify in detail the resulting modifications done in the manuscript text (marked with *italic* text) and/or R package. To facilitate reading, our responses are marked in blue.

**Response to Reviewer 1**

**1)** I can see the potential of this contribution to the field that may prove a regularly used package by researchers, programmers and decision makers. The manuscript is clear and well written, the Figures are informative and it fits well with the journal’s scope. The package worked generally well when using default parameters, however when using the custom settings (particularly for probabilities flowing from host seeking to oviposition), the example custom estimates did not work and neither did a range of other probabilities (see minor comments). It would be useful to determine the range of probabilities that do work so that the user selection could be restricted within this – especially wherever data are available to support the choice (for example, falciparum, to my knowledge only infects humans so Kvi: Proportion of susceptible mosquitoes that become infected after biting, for animals, has to take the value 0 and the user should be restricted to choosing this estimate). The results that could be produced in the absence of this guidance are a little concerning, so I think putting in these restrictions, potentially offering default ranges for mosquitoes other than gambiae for def_host_params() would be very useful indeed.

We thank the reviewer for the positive evaluation and for identifying the issue with the possible ranges of the probabilities in the example from the documentation. We agree that a problem arises if the modelled mosquito survival (equivalent to the sum of the products of all the probabilities of the mosquito feeding cycle across all host types in absence of interventions) is much lower than the provided parous rate. In this case, the transition probabilities provided by the user are too low.

Briefly, the parous rate, $M$, is equal to probability that a mosquito survives an entire feeding cycle, as defined in the model equations from (Chitnis, Smith et al. 2008):

$$M= P_{f}=\frac{\sum_{i=1}^{n} P_{A^{i}}P_{B^{i}}P_{C^{i}}P_{D^{i}}P_{E^{i}}}{1-P_{A}}$$

where:

n = number of types of hosts (humans and animals)

$P_{A^{i}}$ = probability that a mosquito has found a host of type $i$ and has ended the host seeking stage

$P_{B^{i}}$= probability that a mosquito bites a host of type $i$ after encountering a host of type $i$

$P_{C^{i}}$= probability that a mosquito finds a resting place after biting a host of type $i$

$P_{D^{i}}$= probability that a mosquito survives the resting phase after biting a host of type $i$

$P_{E^{i}}$= probability that a mosquito lays eggs and restarts host-seeking after biting a host of type $i$

$P_{A}$= probability that the mosquito stays in the host seeking stage, equal to ${1-A}_{0}$ where $A_{0}$ is the sac rate, which is one of the input bionomics parameters.

The equation above is used to derive the probabilities $P_{A^{i}}$ in absence of interventions as follows (according to Equations 14 and 15 from the Additional File 2 in (Briët, Impoinvil et al. 2019):

- For human hosts:

$$P_{A^{1}}=\frac{A_{0}M\chi\zeta_{2}P_{B^{2}}P_{C^{2}}}{P_{B^{1}}P_{C^{1}}\left( \left( \chi P_{D^{1}}P_{E^{1}}+(1-\chi)P_{D^{2}}P_{E^{2}} \right)\zeta_{2}P_{B^{2}}P_{C^{2}} \right)}$$

- For animal hosts:

$$P_{A^{2}}=\frac{A_{0}M(1-\chi)}{\left( \chi P_{D^{1}}P_{E^{1}}+(1-\chi)P_{D^{2}}P_{E^{2}} \right)\zeta_{2}P_{B^{2}}P_{C^{2}}}$$

where:

$A_{0}$ = sac rate, an input bionomics parameter

$M$ = parous rate, an input bionomics parameter

$\chi$ = human blood index, an input bionomics parameter

$\zeta_{2}$ = relative availability of animal hosts, equal to 1

$P_{B^{i}}, P_{C^{i}},P_{D^{i}},P_{E^{i}}$ = transition probabilities between the different states of the feeding cycle in absence of interventions (see above), input parameters provided by the user

Therefore, for a solution to exist, the two equations above need to give outputs between 0 and 1, i.e., the survival reflected by the mosquito bionomics parameters (parous rate, sac rate, human blood index) needs to be in agreement with the modelled survival through the transitions probabilities $(P_{B^{i}}, P_{C^{i}},P_{D^{i}},P_{E^{i}})$ provided by the user.

We have included the above information in a section in the Supplementary Material: “1 Guidelines for Input Parameters in the Package”. This section includes a description of this special case. In addition, information about this special case accompanied by an example has also been added in the package documentation (section “Defining host-specific entomological parameters”).

We have implemented an error message in the package for instances where user-entered values result in this special case: “Simulated mosquito dynamics during the feeding cycle is not reflected by the mosquito bionomics parameters! Consider increasing the values for PBi, PCi, PDi, PEi in the def_host_param() function call.”

We agree that Kvi should be set to 0 for animal hosts for *Plasmodium falciparum*. However, this does not generally apply, e.g. for *Plasmodium knowlesi*. Since the package is not exclusively focused on *P. falciparum*, we allow the user to define this value as appropriate for their specific use case. We have added a note to this effect in the documentation (in the section “Defining host-specific entomological parameters”) and in the Supplementary Material (section 1 Guidelines for Input Parameters in the Package).

We have added in the package data object host_ent_params the default parameters for these transition probabilities in absence of interventions ($P_{B^{i}}, P_{C^{i}},P_{D^{i}},P_{E^{i}}, Kvi)$ for the major vector species *Anopheles gambiae*, *Anopheles funestus* and *Anopheles arabiensis*, following (Chitnis, Smith et al. 2008) and (Briët, Impoinvil et al. 2019). In absence of better data, the user can choose the same values for other mosquito species.

**2)** I have one other major concern for the authors to consider and otherwise very minor comments and suggestions for future directions.

The **major concern** is the presentation of impacts from interventions without any apparent validation. The uncertainty shown is minimal. I think it could be hugely useful to

i) Include some validations: to do this, you could use data from PMI, or RCTs that tracked mosquitoes over time in a baseline year and then into the intervention year. Plotting the cross sectional estimates for reduction in vectorial capacity onto the outputs could inform whether it is sensible to show more uncertainty in the figures, and reassure the user on inferring from these analyses
ii) Include explicit warnings that these results are not validated and are dependent on the assumptions of the underlying mechanisms.

We agree with the reviewer that the validation of the resulting intervention impacts obtained with the package is important. One key purpose of the AnophelesModel package is to facilitate carrying out validations of the various intervention models. Several models for IRS and LLIN effects included in the package have been validated in previous analyses. One example is an analysis where the “IRS10” parameterisation was used in the OpenMalaria model. The resulting simulations captured accurately the serology-based observed dynamics in malaria prevalence in a trial cohort from the Rachuonyo South District in Kenya (Fig 5 in (Stuckey, Stevenson et al. 2012)). A validation of the “IRS03” and “LLIN02” intervention parameterisations has been conducted in (Korenromp, Mahiané et al. 2016) where OpenMalaria simulations using these parameterisations reproduced the malaria prevalence and relative risk using data from three cluster-randomized ITN trials (validations shown in (Korenromp, Mahiané et al. 2016), precisely in Fig 4 and in *Additional File 3. Simulation input assumptions and calculations on statistically predicted burden reductions for ITN trials*). The model for the effect of house screening together with the other available IRS and ITN model parameterisations have not been validated yet.

We have reflected the information above in the package by displaying a message about the source of the model (publication reference) and existing (or missing) validation, whenever the user selects an existing intervention model from the package database. Furthermore, the following paragraph has been added to the Discussion:

*“The package can help parameterize various intervention models for locally specific conditions in trial settings, and thus check and validate the performance of these models in simulating the trial results. This can be done using malaria survey data or randomized control trial data, as demonstrated in previous studies for several intervention models included in the package (Stuckey, Stevenson et al. 2012, Korenromp, Mahiané et al. 2016). The package informs the user about the availability or absence of previous validation whenever the user selects an intervention model from the database.”*

Currently, the package outputs account for the uncertainty in the mosquito bionomics parameters. The section “4. Incorporating parameter uncertainty” from the Supplementary Text S1 describes how the user can incorporate the uncertainty for other parameters in the analysis, when the uncertainty information is available.

**3** With this in mind, I am not sure it is sensible to include house screening immediately, unless you can validate the estimated impacts. In my experience, screening of houses can lead to very variable outcomes depending on the house style, the degree of screening, the type of materials used for netting, the inclusion or not of insecticide on this material etc… so to show simply a linear reduction in vectorial capacity may not be too useful and may detract confidence in using the otherwise helpful package.

As mentioned in the main manuscript (section *Effects of house screening included in the package*), the house screening parameterisation is based on published work from (Briët, Impoinvil et al. 2019) based on data from Belize (Wagman, Grieco et al. 2015) and Gambia (Kirby, Ameh et al. 2009). These studies found similar reductions in mosquito house entry following house screening (54% and 59%, respectively). Consequently, the house screening model included in the package uses a reduction of 59% to the availability of humans to mosquitoes which results in a linear reduction in vectorial capacity. This model has not been validated yet with randomized control trial data and we have included a warning in the package whenever the user chooses this model.

There is good flexibility in the package and the instructions are very helpful and comprehensive (with the already mentioned concerns over ranges for probabilities that could be restricted).

We thank the reviewer for the positive feedback.

**Minor comments**

**4** The online info needs updating for the now published Lemant et al, - currently referenced as “in preparation”: [AnophelesModel Documentation • AnophelesModel (swisstph.github.io)](https://swisstph.github.io/AnophelesModel/articles/AnophelesModel.html#introduction) in the Mosquito bionomics section of the database explanation

We have updated the citation in the documentation.

**5** There is Anopheles gambiae and Gambiae

There is Anopheles funestus and funestus

Presumably these are the same species (complex?) – when the Bayes fitting was performed, were these grouped? The values for these are quite different (e.g. see Chi)

We apologise for the confusion. Indeed, the package includes estimated bionomics parameters (data object vec_ent_param) for individual *Anopheles* mosquito species, but also for complexes regrouping several species. For example, Gambiae refers to the Gambiae complex, while *Anopheles gambiae* refers to the mosquito species. The Gambiae complex includes *Anopheles gambiae*, *Anopheles arabiensis*, *Anopheles melas*, *Anopheles merus*, and other mosquitoes whose exact species was not identified but belong to the complex. Therefore, it is expected that the bionomics parameters will differ between the individual species and the complex. We have modified the naming convention in the package, using “*Anopheles gambiae”* for the mosquito species and “Gambiae complex” for the complex (same format for other species and complexes) and we have added a note about this in the package documentation (section AnophelesModel database > Mosquito bionomics).

**6** As Zeta (relative availability of different non-humans hosts) is 1 for the full data base is this indicative of a parameter you would like to estimate in the future but currently cannot gain sufficient data for? Or does 1 indicate something specific?

Zeta is a parameter which is currently not measured by entomological surveillance but which can vary for example in rural versus urban settings. Currently, we assume that there is no difference in the relative availability. We have added this information in the Supplementary Material Section 1 Guidelines for Input Parameters in the Package.

The activity patterns data base is hugely useful and disaggregated. Thank you

**7** Running through the help documentation, the function

plot_impact_species(impacts, "VC_red")

worked only when probabilities were high enough when using

model_params = build_model_obj(custom_ent_params, custom_host_params, custom_activity_obj, host_pop)

From the instructions:

For using a different, custom parameterisation, first we need to build a data frame with the same structure as host_ent_params:

species_name = "Anopheles example"

host = [c](https://rdrr.io/r/base/c.html)("human", "animal")

PBi = [c](https://rdrr.io/r/base/c.html)(0.67, 0.44)

PCi = [c](https://rdrr.io/r/base/c.html)(0.33, 0.434)

PDi = [c](https://rdrr.io/r/base/c.html)(0.647, 0.344)

PEi = [c](https://rdrr.io/r/base/c.html)(0.672, 0.214)

Kvi = [c](https://rdrr.io/r/base/c.html)(0.52, 0.314)

custom_params_tab = [cbind.data.frame](https://rdrr.io/r/base/cbind.html)(species_name, host, PBi, PCi, PDi, PEi, Kvi)

Afterwards, we can provide this data frame to the relevant function for defining the host parameters:

custom_host_params = [def_host_params](https://swisstph.github.io/AnophelesModel/reference/def_host_params.html)(mosquito_species = species_name, host_table = custom_params_tab)

#> [1] "Using host-specific entomological parameters provided by the user."

Using the following: model_params = build_model_obj(custom_ent_params, default_host_params, custom_activity_obj, host_pop)

Worked as per the example.

I tried various combinations of the probabilities, many gave very odd outputs in the plot

p = [plot_impact_species](https://swisstph.github.io/AnophelesModel/reference/plot_impact_species.html)(impacts, "VC_red")

[plot](https://rdrr.io/r/graphics/plot.default.html)(p)

Generally, as long as the human probabilities are high enough, there is a reasonable return. It would be sensible to explore the range in the probabilities that give reasonable solutions and restrict users to selecting from these ranges. Or, set assumed probabilities for major vectors to guide the user. As noted Kvi for animals should surely be 0 for *falciparum*. Should input probabilities here always be assumed in the absence of interventions?

The input probabilities defined with def_host_params() are in absence of interventions as specified in the documentation section “Host-specific entomological parameters”. We have corrected the example described by the Reviewer and described in detail in the answer to Comment 1 above the resulting modifications in the manuscript and in the package documentation about when the related exception cases arise. A message has been added upon call of the def_host_params() function when the user input parameters lead to such an exception case. We have included an example of such an exception case in the package documentation (section “Step 2: Defining host-specific entomological parameters”).

In addition, we have added in the data object host_ent_param default values for the transition probabilities for the major vector species *Anopheles gambiae*, *Anopheles funestus* and *Anopheles arabiensis*, following (Chitnis, Smith et al. 2008) and (Briët, Impoinvil et al. 2019). This information is also reflected now in the package documentation (section “Host-specific entomological parameters”).

**8** Why can the user not alter PAi?

As mentioned in the answer to Comment 1 above, the probability $P_{A^{i}}$ in absence of interventions is not a user input, it is calculated according to Equations 14 and 15 as described in the Additional File 2 in (Briët, Impoinvil et al. 2019):

- For human hosts:

$$P_{A^{1}}=\frac{A_{0}M\chi\zeta_{2}P_{B^{2}}P_{C^{2}}}{P_{B^{1}}P_{C^{1}}\left( \left( \chi P_{D^{1}}P_{E^{1}}+(1-\chi)P_{D^{2}}P_{E^{2}} \right)\zeta_{2}P_{B^{2}}P_{C^{2}} \right)}$$

- For animal hosts:

$$P_{A^{2}}=\frac{A_{0}M(1-\chi)}{\left( \chi P_{D^{1}}P_{E^{1}}+(1-\chi)P_{D^{2}}P_{E^{2}} \right)\zeta_{2}P_{B^{2}}P_{C^{2}}}$$

where:

$A_{0}$ = sac rate, an input bionomics parameter

$M$ = parous rate, an input bionomics parameter

$\chi$ = human blood index, an input bionomics parameter

$\zeta_{2}$ = relative availability of animal hosts, equal to 1

$P_{B^{i}}, P_{C^{i}},P_{D^{i}},P_{E^{i}}$ = transition probabilities between the different states of the mosquito feeding cycle in absence of interventions, input parameters provided by the user

This information is now included in the package documentation and in the Supplementary Material Section 1 Guidelines for Input Parameters in the Package.

**9** Perhaps add in a Box to the main manuscript to define the main aspects of mosquito life cycles for the reader (human blood index, sac rate etc…)

We have added the following Box in the main manuscript:

***Box1: Key aspects of the mosquito feeding cycle***

*Every day, mosquitoes emerge from breeding sites. After mating, female mosquitoes enter a feeding cycle characterized by a set of key consecutive states (Fig 1 middle panel). First, mosquitoes begin their search for a host to take a blood meal from, entering the “Host Seeking” state A. During this search, they can either die or land on a host of type* $i$ *(human or animal) and start feeding, transitioning to the “Feeding” state B with probability* $P_{A^{i}}$*. While feeding, they can either die, or successfully complete their blood meal and start searching for a resting place, moving to state C with probability* $P_{B^{i}}$*. They can die while searching for a resting place, or find one and enter the “Resting” state D with probability* $P_{C^{i}}$*. If they survive resting, they return to the breeding site to lay their eggs, entering the “Ovipositing” state E with probability* $P_{D^{i}}$*. Surviving oviposition, they re-enter the cycle and start searching for a host again with probability*$P_{E^{i}}$*. Each vector control intervention affects mosquitoes at specific stages in the feeding cycle (Fig 1 middle panel).*

*Several field-measurable bionomics parameters provide insights into the success of mosquitoes in completing their lifecycle. These parameters include:*

- *The parous rate, which indicates the proportion of host-seeking mosquitoes that have laid eggs at least once.*
- *The human blood index, representing the proportion of blood meals that mosquitoes obtain from humans.*
- *The sac rate, corresponding to the proportion of mosquitoes that laid eggs on the same day.*
- *The preference of mosquitoes to rest and feed indoors, known as endophily and endophagy, respectively.*

**10** Once the above are considered, I support the publication of this manuscript and look forward to the community making use of the database and informative functions that the team have put together.

Adding in other interventions as next steps makes good sense. Providing some validations to the outputs would be highly worth the effort, and would add support for users, particularly those wanting to make use of the tool to support decisions on procurement and deployment of various intervention options. Guiding the user by placing some data-informed restrictions on input values would be sensible. Another useful step would be to enable the combination of interventions to be explored. In addition, providing some guidance on how you would suggest multiple species to be considered. Perhaps you could look at weighted averages for estimates that represent species composition. Again, with all these steps validation would prove a worthy exercise.

We thank the reviewer for the positive evaluation of our work and for the suggestions that have strengthened our analysis. We have tried to our best to address the mentioned comments (see above responses and resulting modifications in the manuscript) and hope these address all the reviewer’s concerns.

We have also created a new functionality, which allows combining interventions (explained in the Documentation in the section “Combining interventions” and added an example in the main manuscript in the Results section:

*In a second analysis, for the same mosquito species, we compared the estimated impact of LLINs to other interventions available in the package, namely IRS, house screening, and a combination of LLINs and IRS (Fig 4). For all interventions, the effects for An. gambiae were superior to those for An. farauti. Sections 5 and 6 in the Supplementary Text S1 show how this analysis can be extended to OpenMalaria and to combinations of several mosquito species.*

**Figure 4: Estimated impact of various interventions for An. gambiae (solid curves) and An. farauti (dashed curves).** The impact was estimated for the three types of interventions available in the package (IRS, LLINs and House screening), as well as for the combination of IRS and LLINs. The ribbons correspond to the variation of the reduction in vectorial capacity estimated based on the confidence intervals of the mosquito bionomics parameters.

We thank the Reviewer for the suggestion about considering multiple species. The package currently estimates the effects of vector control interventions on the vectorial capacity for a species at a time. Nevertheless, as the Reviewer suggests, it is possible to combine the outputs from several species through a weighted average by using as weights their relative density in a geographical setting. We have added a separate section in the Supplementary Material Section “*5. Considering multiple mosquito species*” to detail this and referenced it in the main manuscript as shown in the paragraph mentioned above.

**Response to Reviewer 2**

This package seems like a valuable contribution to the field, providing accessible databases of important mosquito, human, and intervention parameters, and integrating them into a user-friendly package to estimate the location-specific impacts of mosquito-borne disease intervention strategies. The package documentation linked in the article is easy to follow and thorough, and the article submitted here provides a helpful overview of the package functionality along with a specific use-case example. I believe this publication would be a valuable contribution to the field, made stronger by a few simple changes to help potential users understand what data they provide and how it will be utilized. These changes are detailed below.

We thank the reviewer for the positive evaluation of our work. We have strived to address the comments below.

Content Comments:

**11** Abstract lines 23-25 do not give me a clear understanding of what this package does. Borrowing the phrasing from your GitHub documentation, perhaps you could rephrase this to: “AnophelesModel is an online, open-access, R package that quantifies the impact of vector control interventions using mosquito species and location-specific characteristics.”

We thank the reviewer for the suggestion, we have rephrased accordingly.

**12** Figure 1: I do not follow the model of mosquito feeding cycle and could use a description of what states are included (A-E) in the caption, or perhaps more clarity in the figure. I see 5 state categories (A-E) but only four category descriptions (host seeking, feeding, resting, ovipositing). The fifth state of “searching for a resting place” is specified in line 106 but should be included here for clarity.

We have updated the figure to include the missing state and reflect more accurately the model which was published in (Chitnis, Smith et al. 2008):

We have also modified the figure caption accordingly:

*“****Figure 1: Overview of the AnophelesModel R package and its components.*** *The package integrates various types of input data (first panel) to parameterise an existing model of the mosquito feeding cycle (middle panel, schematic adapted from (Chitnis, Smith et al. 2008)) This model represents the feeding cycle states with letters A through E and transition probabilities P_A_^i^ – P_E_^i^ between consecutive states for a host of type i (human, animal hosts). The dotted, grey arrows indicate that mosquitoes can die at each stage. Blue lightning symbols indicate the transition probabilities affected by the vector control interventions included in the package. This model is used to infer the species-specific effects of vector control interventions, including the decay of these effects over time and their impact on vectorial capacity (third panel).”*

Furthermore, we have added Box 1 in the main manuscript to describe the key aspects of the mosquito feeding cycle and the related field-measurable metrics (see above response to the previous Reviewer, Question 9).

**13** Figure 1: What are the different colored lines in the “Reduction in vectorial capacity” subfigure? If they are meant to represent two different control measures, perhaps label them as such.

They represent indeed two interventions. We have added more explanatory information on the figure (see previous answer).

**14** Design and Implementation section (line 91): Specify exactly what data the user needs to input for a model simulation.

We have added the following paragraph at the location specified by the Reviewer: *“AnophelesModel estimates the species- and geographic-specific impact of vector control interventions by integrating multiple layers of input data (Fig 1). Users can select input data on mosquito bionomic characteristics, mosquito and human activity patterns, and the effects of interventions (cf. Sect. 1 in Supplementary Text S1) from a curated database included in the package, or input their own data.”*

In addition, we have added a dedicated section in the Supplementary Material referenced in the above paragraph, “*1.	Guidelines for input parameters in the package*”, which includes detailed specifications of the input parameters and key considerations when defining them.

**15** Line 95: Define vectorial capacity here instead of on line 110.

We have moved the definition of the vectorial capacity at the indicated place.

**16** Line 109-111: It would be helpful to understand how intervention effects reduce model transition probabilities. Do mosquitoes just stay in each state for longer due to interventions? Or does it reduce the number of mosquitoes the model is initialized with?

Following interventions, mosquitoes die at different stages (each intervention affects mosquitoes at specific stages, Fig 1 middle panel). Using already published models that we include in the package (described in the Supplementary Material, Section 2), we calculate the proportions of mosquitoes that survive to the next stage. Currently the package does not include parameterisations for interventions that reduce the number of emerging mosquitoes (e.g., larviciding). We have added this information in the main manuscript (at the end of the section “Modelling the effects of vector control interventions on the mosquito feeding cycle”) and marked on the model diagram (Fig 1 middle panel) the transition probabilities that are influenced by interventions included in the package.

**17** Line 125: My interpretation of what follows this sentence is that interventions may deter mosquito-human interaction (skipping feeding state and potentially increasing death rate) or invoke mosquito death at some rate either prior to or after feeding. These impacts cannot be expressed as simple reductions in the rate parameters of Figure 1, middle panel, and I think the figure would be more helpful if the actual intervention effects were better captured! Extending your model diagram from https://swisstph.github.io/AnophelesModel/articles/AnophelesModel.html with arrows to indicate the impacts of intervention measures would be helpful for understanding.

We have updated Fig 1 accordingly and marked the transition probabilities that are influenced by the interventions included in the package. We have also updated the model diagram from the documentation to this figure. In the Supplementary Material, section “2. Modeling the effects of vector control interventions” describes in detail the methods and published models used to calculate the impacts of the different interventions on the transition probabilities of the feeding cycle.

**18** Figure 2: The first caption sentence was not clear to me. Perhaps rephrase to “The package provides (A) entomological parameters, (B) mosquito biting patterns, (C) human activity patterns, and (D) intervention properties, which can be used to…”

We have modified the caption as suggested.

**19** Figure 2: I do not see the gray area mentioned for panel (B).

This could have happened due to the file format. We have replaced the figure with a high-resolution version.

**20** Figure 2: Add a description for panel C as included for all other subplots.

We have updated the caption of Figure 2 to include descriptions of all panels:

*“The package provides (A) entomological parameters, (B) mosquito biting patterns, (C) human activity patterns, and (D) intervention properties, which can be used to parameterise an entomological model of the mosquito feeding cycle. Examples are provided for An. gambiae and An. farauti in Kenya and Papua New Guinea (PNG) settings, respectively. In panel (A), the arrows indicate the bars corresponding to the two mosquito species. In panel (B), hourly biting rates are shown for both species, while the grey area highlights the time when people sleep under a net. Panel (C) displays the hourly proportions of people located indoors and outdoors for each geographical setting. Panel (D) summarizes the observed variation in physical properties of LLINs in a Kenya-like setting (9) for each semester (every 6 months) during 3 years. Data sources of all data types are specified in the “Design and Implementation” section.”*

**21** Figure 2D: What is a “semester” on the horizontal axis? Perhaps include this in the caption.

We have included the information in the caption (see previous answer).

**22** Figure 4: While I know this example is mostly included to showcase how model functionality and the ease of pipelining to OpenMalaria, it may be more interesting to show an example where the two treatment strategies result in drastically different impacts. This could likely be achieve by simply simulating multiple treatment rounds in OpenMalaria.

We thank the reviewer for this suggestion that has led to a more interesting example. We have added two deployments of ITNs at 40% coverage in both settings to emphasize the difference in the obtained impact of LLINs deployment. The text describing this example and the figure (now Fig S1) have been updated accordingly. We have moved the OpenMalaria example in the Supplementary Material, in the section “*6. Using AnophelesModel to parameterize OpenMalaria*” as asked by Reviewer 3 (answers to Comment 27 below).

***Figure S1: Simulation of the impact of LLINs deployment in OpenMalaria.*** *XML snippets produced by AnophelesModel were used in OpenMalaria to model the entomology and effects of LLINs deployments in Kenyan-like and PNG-like settings and to simulate all-age prevalence. Two deployments of LLINs were simulated in January 2023 and 2025 (dashed lines). The prevalence during the simulated years (2021-2029) is displayed for both settings. The shaded areas around the curve show the uncertainty in the estimated prevalence.*

**23** Grammar Comments:

Line 23: Remove comma after “open-access”

Line 27: remove word “on”

Line 164: “using” -> “use of”

Line 168: “are adjusted by multiplying them by the…” -> “are scaled by the…”

Line 177 and 184: Add comma before “and” in compound sentence.

Line 189: Add “with” after “associated”.

Thank you for pointing out the grammar errors and typos. We have corrected them accordingly.

**Response to Reviewer 3**

The manuscript introduces an R package specifically crafted to process input data on vector bionomics, facilitating the generation of species-specific impacts on the carrying capacity of vector control interventions. It stands as a solid, well-written manuscript, and fits well within the broader scientific context of malaria modeling and vector research.
The R package offers a valuable contribution to the field, particularly in its interaction with a precompiled, or user-defined, database allowing to project impact on vectorial capacity for different mosquito species and vector control interventions.

In consideration of a wider malaria research community, and to enhance its value beyond OpenMalaria users, the manuscript could highlight the functionality of its three main components more prominently.

**24** The core functionality of the model is the estimation of species and geographic-specific impact of vector control interventions, which is described in main text and supplementary file. This direct output of the software is illustrated in Figure 2. Conversely, the simulated health impact, which stems from external malaria model simulations (depicted in Figure 3), is not. The authors might therefore consider moving Figure 3 and description of OpenMalaria to the supplement, to allocate more space for elaboration on the various options for estimating intervention impact. For instance, including all three interventions available, such as IRS and house-screening.

We thank the Reviewer for this suggestion. We have moved the analysis part with OpenMalaria and the related Figure to the supplement and have replaced it with an additional example of using all interventions available in the package, including a combination of LLINs and IRS (detailed also in the answer to Reviewer Comment 10 above).

**25** Relatedly, in the current format, the manuscript might also inadvertently imply that the ready-to-use outputs could be utilized for established transmission models, although this applies solely to OpenMalaria. Hence the title and heading might appear overstated.

The outputs of the model are not exclusive to OpenMalaria. Our model produces vectorial capacity estimates which can be directly included in any compartmental model of malaria dynamics as done in previous publications such as (Briët, Impoinvil et al. 2019, Champagne, Gerhards et al. 2022, Champagne, Gerhards et al. 2023). We have adapted the content of the section “Interfacing with models of malaria transmission dynamics” from the main manuscript to reflect this information:

*“The AnophelesModel package generates vectorial capacity estimates that can be seamlessly integrated into any compartmental model of malaria dynamics. This approach has been successfully utilized in previous studies, such as: (Briët, Impoinvil et al. 2019, Champagne, Gerhards et al. 2022, Champagne, Gerhards et al. 2023). In addition, the package estimates the decay of intervention effects over time and generates parameterizations of vector control components which may be used for running simulations with the OpenMalaria model. Specifically, AnophelesModel contains functions for producing formatted entomology and intervention input for the OpenMalaria model.”*

Suggestions for improvements the authors might consider include:
**26** 1) Elaborate more on the generic utility of the model, (i,e, how in R to interact with the functionalities described in the supplement , how to work with the input data, or description of effects generated for the two other types of interventions available) would be of greater interest for the broader community.

We thank the Reviewer for this suggestion. We have included more details about choosing the intervention inputs in the Supplementary Material Section 1 “*Guidelines for Input Parameters in the Package*”. We have added in the documentation as well as in the main manuscript an additional example displaying the vectorial capacity with other interventions available in the package and with intervention combinations (Figure 4 presented in the reply to Comments 24 and 10 above). The documentation provides step-by-step examples to guide users on how to interact with the package and utilize its functionalities. The importance of this documentation is highlighted several times in the main manuscript.

**27** 2) Move the example "Interfacing AnophelesModel with models of intervention impact" to the supplement, as the simulated health impact is not a direct output of the software presented in the Article. The space can instead be used to address 1)
We have moved the concerned section to the supplement and added an additional example as suggested (cf. previous answer).

Some minor suggestions and comments:
**28** - Aligning or integrating the flowchart presented in the manuscript with the programmatic flowchart outlined in the R package documentation. Notably, in the manuscript intervention effect is included as ‘data’ (which one might interpret to be an input), whereas in the package documentation it is presented as a part of the entomological model component.

We have updated the flowchart in the package documentation to reflect the sequence of the key functions. The intervention effects are indeed inputs, but they are defined after the entomological model has been initialized as they interact with the model. On the diagram, we have renamed the model component accordingly, to reflect this initialization step and have included the updated diagram in the documentation:

**29** - The methods section would benefit from adhering to the same structural framework as depicted in the flowchart (Figure 1),i.e. describing the three data types first, then the Entomological model of the mosquito feeding cycle and vectorial capacity

We have restructured the Methods section to follow the same order as in Fig 1.

**30** - Figure 2D and Fig 3: A) both describe the LLIN decay however they use different units and description in the x-axis, and the text provides an example of decay in years. This could be considered to be aligned, i.e. using semester also in Fig 3A.

We have aligned the two figures by marking the individual semesters on the decay plots in Fig 3:

**Figure 3: Estimated effects of LLINs deployment for An. gambiae and An. farauti.** Mosquito, human and intervention data are combined in the AnophelesModel package to estimate the different types of intervention decay throughout time **(A)**, as well as the resulting mean reduction in vectorial capacity for varying LLINs deployment coverages (here equivalent to LLINs usage) **(B)**. The time units in panel **(A)** are defined by 100 equally distanced interpolation points across the duration of the interventions (i.e., 3 years for LLINs, with dotted lines marking each semester). The ribbons in panel **(B)** correspond to the variation of the vectorial capacity estimated based on the confidence intervals of the mosquito bionomics parameters (details on uncertainty propagation provided in the Supplementary Material Sect. 4).

**31** - Figure 4: The Kenya and PNG settings in the figure are missing the ‘like’. Given the high heterogeneity of vector dynamics within a country using the region names would be (i.e. Kisumu) would be clearer. For most clarity, author might consider labelling the outcome directly by the vector species, since only one vector species was used while in geographic areas often multiple vectors occur. In the two simulations for Kenya and PNG, the input EIR was set to the same level, not ‘similar’ (i.e. L286, L311)

We appreciate the reviewer's suggestion, which has helped clarify the results in this figure. We have updated the legend to label the outcome by vector species and modified the example to accommodate the comment from Reviewer 2 (cf. answer to Comment 22). The figure together with the section about OpenMalaria have been moved to the supplementary materials, as suggested by the Reviewer. Additionally, we have corrected the text to accurately state that the EIR is indeed the same, not just similar.

**32** - The inclusion of syntax and output examples would be beneficial for this type of article.

Due to the word limit for the main manuscript, we have not been able to include R code in the main manuscript. Nevertheless, to provide additional complementary information, we have incorporated a section in the Supplementary Material “1 Guidelines for Input Parameters in the Package”. We have also added in the main manuscript an example showing the impact of combinations of interventions. As mentioned in the manuscript (section *Availability and Future Directions*), a comprehensive package documentation with detailed examples is available at:

<https://swisstph.github.io/AnophelesModel/articles/AnophelesModel.html>

**33** - It's important to address how insecticide resistance is considered, or can be factored in when utilizing the package to obtain intervention effect sizes. While experimental hut trial and cluster randomized trial data are used to parameterize effects, it's crucial to clarify whether resistance levels in the area are accounted for in the analysis, or whether all effects generated apply to fully susceptible mosquitoes.

Several intervention parameterisations available in the package are derived from locations where mosquitoes presented insecticide resistance, therefore resistance is accounted for in the effects. We have added information about whether a parameterization is considering resistance or not in Supplementary Table S1 as well as in the package, in the intervention summary object (interventions_param$interventions_summary). This information has also been reflected in the package documentation (Section “Step 5: Defining interventions effects”).

**34** - A potential user of the package might also be interested in whether the vector model can produce intervention effect for the combinations of LLINs and IRS , which likely affets the probabilities in host-related entomological parameters hence the reduction in vectorial capacity?

We thank the reviewer for this observation. We have added this new feature to the package and presented it in the manuscript, including an example in the new Fig 4 (see responses to Reviewer comments 10, 24) and the section “Combinations of interventions” in the package documentation.

**References**

Briët, O. J., et al. (2019). "Models of effectiveness of interventions against malaria transmitted by Anopheles albimanus." Malaria journal **18**(1): 1-12.

Champagne, C., et al. (2022). "Using observed incidence to calibrate the transmission level of a mathematical model for Plasmodium vivax dynamics including case management and importation." Mathematical Biosciences **343**: 108750.

Champagne, C., et al. (2023). "Quantifying the impact of interventions against Plasmodium vivax malaria: a model for country-specific use." medRxiv: 2023.2002. 2010.23285652.

Chitnis, N., et al. (2008). "A mathematical model for the dynamics of malaria in mosquitoes feeding on a heterogeneous host population." Journal of Biological Dynamics **2**(3): 259-285.

Kirby, M. J., et al. (2009). "Effect of two different house screening interventions on exposure to malaria vectors and on anaemia in children in The Gambia: a randomised controlled trial." The Lancet **374**(9694): 998-1009.

Korenromp, E., et al. (2016). "Malaria intervention scale-up in Africa: effectiveness predictions for health programme planning tools, based on dynamic transmission modelling." Malaria journal **15**: 1-14.

Stuckey, E. M., et al. (2012). "Simulation of malaria epidemiology and control in the highlands of western Kenya." Malaria journal **11**: 1-14.

Wagman, J. M., et al. (2015). "The field evaluation of a push-pull system to control malaria vectors in Northern Belize, Central America." Malaria journal **14**(1): 1-11.
